# Promoter hypomethylation, especially around the E26 transformation-specific motif, and increased expression of poly (ADP-ribose) polymerase 1 in BRCA-mutated serous ovarian cancer

**DOI:** 10.1186/1471-2407-13-90

**Published:** 2013-02-26

**Authors:** Fang-Fang Bi, Da Li, Qing Yang

**Affiliations:** 1 Department of Obstetrics and Gynecology, Shengjing Hospital, China Medical University, 110004, Shenyang, China; 2 Department of Physiology, Institute of Basic Medical Sciences, China Medical University, 110001, Shenyang, China

**Keywords:** PARP1, ETS, BRCA mutation, Ovarian cancer, Methylation

## Abstract

**Background:**

Poly (ADP-ribose) polymerase 1 (PARP1) overexpression plays a critical role in ovarian cancer progression and the clinical development of PARP1 inhibitors to treat BRCA-mutated ovarian cancer has advanced rapidly. However, the mechanism regulating PARP1 expression remains unknown. Alterations in gene expression mediated by promoter methylation are being increasingly recognized and have frequently been reported in ovarian cancer. We therefore investigated the methylation status of the PARP1 promoter region and its correlation with PARP1 expression in BRCA-mutated ovarian cancer.

**Methods:**

DNA from BRCA-mutated serous ovarian cancer samples and adjacent normal ovarian tissues were analyzed by bisulfite sequence using primers focusing on the CpG island in the promoter region of PARP1. Expression levels of PARP1 were assessed by immunohistochemistry and real-time PCR.

**Results:**

Serous ovarian cancer tissues displayed decreased DNA methylation in the promoter region of PARP1 compared to normal tissue, and methylation intensity correlated inversely with PARP1 mRNA levels. More importantly, E26 transformation-specific (ETS) defined CpG sites were significantly less methylated in ovarian cancer samples.

**Conclusions:**

These results indicate that hypomethylation of the promoter region, especially around the ETS motif might play a role in the upregulation of PARP1 expression in the progression of ovarian cancer.

## Background

Ovarian cancer is the most lethal gynecological malignancy in the United States [1]. To date, although the exact cause of ovarian cancer is not clear, BRCA1 and BRCA 2 mutations are the only known causes of hereditary ovarian cancer [2]. In 2005, two pivotal studies showed that BRCA-deficient cells were especially sensitive to chemical inhibitors of poly (ADP-ribose) polymerase (PARP), which plays a critical role in single-stranded DNA break repair, presumably due to the lack of homologous recombination-dependent DNA repair [3,4]. These findings have raised significant concerns about PARP and PARP inhibitors in ovarian cancer. Recently, emerging evidence has indicated that PARP expression is frequently upregulated in ovarian cancer and is related to worse overall survival [5,6]. However, little is known about the mechanisms regulating PARP gene transcription. In mammals, methylation of cytosine in CpG dinucleotides (especially those located in promoter regions) is an important feature regulating gene expression [7]. Notably, Several lines of data have suggested that PARP1 promoter methylation is involved in the regulation of nano-SiO2-induced decrease of PARP1 expression in HaCaT cells [8] and benzene-induced decrease of PARP1 expression in lymphoblastoid cell line F32 [9]. Our present study is the first to analyze DNA methylation patterns in the PARP1 promoter region in BRCA-mutated ovarian cancer, showing that abnormal hypomethylation of this promoter, especially around the E26 transformation-specific (ETS) motif, may be responsible for PARP1 overexpression.

## Methods

### Patients

This study was approved by the Institutional Review Board at China Medical University. Serous ovarian cancer patients were enrolled between 2010 and 2012, and all patients gave informed consent. Fresh tumor samples, adjacent normal ovarian tissues and blood samples were obtained at the time of primary surgery before any chemotherapy or radiotherapy. Hematoxylin and eosin staining of the samples for histopathological diagnosis and grading were determined by three staff pathologists using the World Health Organization criteria. All patients were screened for BRCA1 and 2 mutations by multiplex polymerase chain reaction with complete sequence analysis as previously reported [10-12]. Briefly, genomic DNA isolated from peripheral blood leukocytes were amplified using specific primers and the amplified products were then sequence analyzed. At the end, ten patients were determined to have a BRCA-mutation. Their characteristics are given in Additional file 1.

### Real-time quantitative PCR

Total RNA was extracted from the tumor and normal ovarian tissues using Trizol reagents (Invitrogen, Carlsbad, CA) according to the manufacturer’s protocol. It was then reverse transcribed using the PrimeScript RT Master Mix kit (TaKaRa, Dalian, China) and amplified by SYBR Premix Ex TaqTM II (TaKaRa) in a Roche LightCycler 2.0 instrument (Roche Diagnostics, Mannheim, Germany). The specific primer sequences were as follows: PARP1: 5^′^- GAGTCGGCGATCTTGGACC -3^′^ (F) and 5^′^- TGACCCGAGCATTCCTCG -3^′^ (R); GAPDH: 5^′^- AGGTGAAGGTCGGAGTCA -3^′^ (F) and 5^′^- GGTCATTGATGGCAACAA -3^′^(R). GAPDH mRNA was amplified as an internal control for normalization of each sample. The PCR cycling conditions were: 45 cycles of 10 s at 95°C, 20 s at 60°C. For all transcripts, the melting curve was obtained after cycling at a stepwise increase in temperature from 55 to 95°C to verify the specificity of amplification. The threshold cycle (Ct) was used to represent the relative mRNA amounts. All samples were analyzed in triplicate using the 2^–ΔΔCT^ method.

### Immunohistochemistry

The standard SP kit (Zhongshan, Beijing, China) was used for immunohistochemical staining. Briefly, serial 4-μm sections were obtained from each paraffin-embedded tissue block. Following deparaffinization and rehydration, sections were subjected to microwave antigen retrieval. The primary antibody was mouse monoclonal anti-PARP1 (1:100; Santa, Cruz Biotechnologies, USA) and the sections were incubated overnight at 4°C with this antibody. 3, 3'-diaminobenzidine (DAB) was used as the chromogen. Nuclei were counterstained with hematoxylin, and slides were dried and mounted. Negative controls were incubated with phosphate-buffered saline (PBS) instead of the antibody. Immunostaining was evaluated by two independent pathologists, blinded to the identity of subject groups. Area quantification was made with a light microscope at a magnification of 400 × and analyzed by Image-Pro Plus 6.0 (Media 2 Cybernetics, USA) using PARP1-positive cells.

### Bisulfite sequencing

Genomic DNA extracted from ovarian carcinoma and normal ovarian tissues with a TIANamp Genomic DNA kit (Tiangen biotech, Beijing, China) was subjected to bisulfite conversion using the EZ DNA Methylation-Direct kit (Zymo research, Orange, USA) following the manufacturer’s instructions, the conversion efficiency was estimated to be at least 99.6%. It was then amplified by nested PCR. After gel purification, cloning and transformation into *E.coli* Competent Cells JM109 (TaKaRa), ten positive clones of each sample were sequenced to ascertain the methylation patterns of each CpG locus. The following primers were used: round I, F: 5^′^- TTGGGATAGAATAATTAAAG -3^′^ and R: 5^′^- AACTTTTCCTACAACATCAA -3^′^; and round II, F: 5^′^- TAGAATAATTAAAGGGGTGG -3^′^ and R: 5^′^- ACAACATCAACAAAACCTT -3^′^. The conditions were as follows: 95°C for 2 min, 40 cycles of 30s at 95°C, 30s at 56°C and 45 s at 72°C, then 72°C for 7 min.

### Statistical analysis

The data are presented as mean ± SD. Statistical differences in the data were evaluated by paired Student’s *t* test, and were considered significant at *P* < 0.05.

## Results

### PARP1 expression was upregulated in BRCA-mutated ovarian cancer

**Figure 1:**
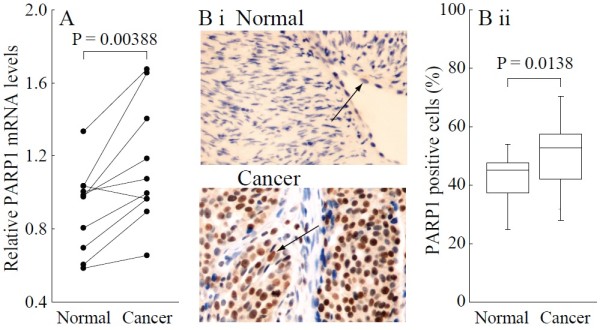
**Overexpression of PARP1 mRNA and protein in BRCA-mutated ovarian cancer. A**, Relative PARP1 mRNA levels were measured by real-time quantitative PCR. **B** i, Sections were subjected to immunostaining for PARP1 in normal and ovarian cancer tissue. Arrow shows positive staining for PARP1 in the nuclei. **B** ii, Summary of the percentages of PARP1-positive cells from the measurements shown in (**B** i). Bar graphs show mean ± SD. Magnification is 400 × .

Real-time PCR and immunohistochemical analysis showed that PARP1 mRNA and protein were overexpressed in ovarian cancer tissues, compared to normal ovarian tissues (*P* < 0.05; each group n = 10; Figure 1A and B).

### BRCA-mutated ovarian cancer tissues displayed a highly hypomethylated ETS motif and promoter region

**Figure 2:**
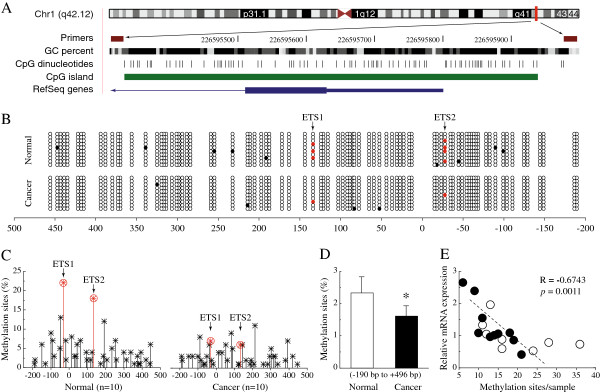
**Hypomethylation of the ETS motif and promoter region of PARP1 in BRCA-mutated ovarian cancer. A**, Location of PARP1 core promoter CpG sites. Genomic coordinates are shown, along with the primer-amplified fragments, GC percentage, location of individual CpG dinucleotides (dashes), CpG island (green bar), and the PARP1 RefSeq gene (exon 1 shown as a blue box and intron shown as an arrowed line). The arrow indicates the transcriptional direction. **B**, Changes of methylation patterns in the core promoter region of PARP1 (each group, n = 10). The circles correspond to CpG sites denoted by the black dashes in (**A**). Closed circles, methylation; open circles, unmethylation. Ten individual clones were sequenced for each sample. Red circles show a methylation of cytosine that located in a CpG within an ETS motif. **C**, Summary of the promoter DNA methylation pattern in ovarian cancer and normal tissues. The y-axis shows the mean methylation sites (each group n = 10). Red circles show a methylation of cytosine that located in a CpG within an ETS motif. **D**, Overall methylation percentage within the promoter region from ovarian cancer and normal tissues (each group n = 10). **E**, Correlation between the relative PARP1 mRNA levels and the total number of methylated sites for each sample. Open circles, ovarian cancer tissues. Closed circles, normal ovarian tissues (each group n = 10). * *P* < 0.05 ovarian cancer *vs.* normal tissues.

To investigate PARP1 transcriptional regulation through epigenetic mechanisms, we assessed the essential regulatory region by analyzing changes in the methylation of important transcription factor-binding sites. As shown in Figure 2B and C, we searched for differences in the DNA methylation pattern between ovarian cancer and normal tissues. Bisulfite sequence analysis showed that ovarian cancer tissues exhibited global hypomethylation, especially around the ETS motif. The total percentage of promoter methylated sites in this region (−190 to +496) was significantly decreased in ovarian cancer compared to normal tissues (*P* < 0.05; Figure 2D). In addition, we noted a significant negative correlation between PARP1 mRNA levels and the total number of methylated sites in both ovarian cancer and normal tissues (Figure 2E).

## Discussion

DNA methylation is an epigenetic phenomenon known to play a critical role in regulating gene expression through interference with the binding of specific transcription factors to recognition sites in promoters [13]. ETS is one of the largest transcription factor families and has a highly conserved DNA-binding domain that recognizes a common sequence motif, 5’-(C/A) GGA (A/T) -3’ [14], which is widely distributed in the PARP1 promoter [15]. The present study showed that BRCA-mutated ovarian cancer displayed a relatively hypomethylated PARP1 promoter, but significantly higher methylation as noted particularly around the ETS motif in normal ovarian tissue. Therefore, we speculate that the important mechanism underlying increased PARP1 expression might be related to the abnormal methylation of CpG sites in the ETS motif, thereby affecting the binding of ETS transcription factors. Previous studies have shown that ETS transcription factors may be key mediators in regulating PARP expression [15]. Furthermore, an increasing amount of evidence suggests that ETS transcription factors are important regulators of the tumorigenic properties of ovarian cancer cells [16] and correlate poor survival in serous ovarian carcinoma [17]. Based on these findings, there are some interesting issues that need to be considered in future studies. PARP1 can enhance DNA methyltransferase 1 (DNMT1) expression by maintaining the unmethylated state of the DNMT1 promoter [18], so it can be predicted that up-regulation expression of DNMT1 may be beneficial in resisting genome-wide demethylation during the progression of ovarian cancer. Moreover, PARP1, as the protein component of chromatin, controls transcription through affecting the chromatin structure [19]. Therefore, PARP1 overexpression may constitute a specific epigenetic mark in BRCA-mutated ovarian cancer. Another report indicated that hypermethylation of the BRCA1 promoter correlated with gene inactivation in sporadic breast and ovarian tumors, as inherited BRCA1 mutations [20]. Thus, it is important for future studies to analyze DNA methylation patterns of the PARP1 promoter in the DNA methylation-associated inactivation of the BRCA1 gene in ovarian cancer.

## Conclusions

Our results indicate that the biological effects of ETS in ovarian cancer might be mediated by the hypomethylated ETS motif, which induces the high expression of PARP1. Therefore, further studies are required to identify how the methylation of ETS affects PARP1 transcription and whether other factors could cooperate with ETS in controlling PARP1 gene expression. If we can clarify the mechanism behind high PARP1 expression from an epigenetic point of view, a more specific epigenetic therapy could be developed for ovarian cancer.

## Abbreviations

PARP: Poly (ADP-ribose) polymerase;ETS: E26 transformation-specific;DNMT1: DNA methyltransferase 1

## Competing interests

The authors declare that they have no competing interests.

## Authors’ contributions

QY conceived the study. FFB and DL carried out data acquisition and interpretation. QY, FFB and DL drafted the manuscript. All authors read and approved the final manuscript.

## Pre-publication history

The pre-publication history for this paper can be accessed here:

http://www.biomedcentral.com/1471-2407/13/90/prepub

## Supplementary Material

Additional file 1 **Clinical characteristics for the 10 BRCA-mutated serous ovarian cancer patients.** (DOC 129 kb) Click here for file
