# Supplementary material for: Promoter hypomethylation, especially around the E26 transformation-specific motif, and increased expression of poly (ADP-ribose) polymerase 1 in BRCA-mutated serous ovarian cancer
Source: BMC Cancer. 2013 Feb 26;13:90. doi: 10.1186/1471-2407-13-90 (PMC3599366; doi:10.1186/1471-2407-13-90)
Supplement: Additional file 1 — Clinical characteristics for the 10 BRCA-mutated serous ovarian cancer patients. (DOC 129 kb) [file 1471-2407-13-90-S1.doc]

**Table 1** Clinical characteristics for the 10 BRCA-mutated serous ovarian cancer patients

| Case | Age | Stage^a^ | Grade^b^ | Gene | Exon | Mutation | AA change | Mutation type^c^ |
| --- | --- | --- | --- | --- | --- | --- | --- | --- |
| 1 | 70 | ⅢC | PD | BRCA1 | 11 | c.2331 T > A | p.Y777X | NS |
| 2 | 47 | ⅢC | MD | BRCA1 | 11 | c.2311 T > G | p.L771V | MS |
| 3 | 54 | ⅢC | PD | BRCA1 | 11 | c.2566 T > C | p.Y856H | MS |
| 4 | 68 | ⅢC | MD | BRCA1 | 11 | c.2612 C > T | p.P871L | MS |
| 5 | 64 | ⅢC | MD | BRCA1 | 11 | c.2612 C > T | p.P871L | MS |
| 6 | 57 | ⅠA | PD | BRCA1 | 11 | c.2566 T > C | p.Y856H | MS |
| 7 | 63 | ⅢC | PD | BRCA1 | 11 | c.2612 C > T | p.P871L | MS |
| 8 | 56 | ⅢC | PD | BRCA1 | 11 | c.2709 T > A | p.C903X | NS |
| 9 | 49 | ⅡB | PD | BRCA2 | 11 | c.3710 C > T | p.A1237V | MS |
| 10 | 52 | ⅡB | MD | BRCA2 | 11 | c.3109 C > T | p.Q1037X | NS |

a: The tumor stages were assessed according to the International Federation of Gynecology and Obstetrics.

b: PD: poorly differentiated; MD: moderately differentiated.

c: NS: nonsense mutation; MS: missense mutation.
